# Development of an online personalized self‐management intervention for men with uncomplicated LUTS

**DOI:** 10.1002/nau.24040

**Published:** 2019-05-20

**Authors:** Marco H. Blanker, Pim Brandenbarg, Bart G.C. Slijkhuis, Martijn G. Steffens, Michael R. van Balken, Petra Jellema

**Affiliations:** ^1^ Department of General Practice and Elderly Medicine University Medical Centre Groningen, University of Groningen Groningen The Netherlands; ^2^ Department of Urology Isala Clinics Zwolle The Netherlands; ^3^ Department of Urology Rijnstate Hospital Arnhem The Netherlands; ^4^ Department of Public and Occupational Health, Amsterdam Public Health Research Institute, Amsterdam UMC Vrije Universiteit Amsterdam Amsterdam The Netherlands

**Keywords:** eHealth, lower urinary tract symptoms, online intervention, self‐management

## Abstract

**Aims:**

To develop an online platform to facilitate evidence‐based self‐management of lower urinary tract symptoms (LUTS) in men.

**Methods:**

Using the PubMed database (search until January 2017) and relevant guidelines, we reviewed evidence for the self‐management of LUTS and identified suitable components for the intervention. Next, we built an algorithm that provided individualized advice based on patient characteristics and symptoms for use on an online platform. Men with LUTS tested the usability of the intervention and provided feedback. Finally, we surveyed urologists and general practitioners to identify potential areas for improvement of the intervention.

**Results:**

We identified nine self‐help interventions from 48 eligible publications. These were as follows: information and education about LUTS, pelvic floor muscle training, bladder training, urethral milking, double voiding, caffeine management, alcohol management, fluid management, and exercise advice. The level of evidence for each item was low due to the paucity of research available. Six men with LUTS reported difficulties understanding and completing the frequency‐volume chart online. The 158 surveyed physicians agreed (≥50% positive ratings) on the inclusion of seven advice items, but not for double voiding and fluid management. Respondents noted that some advice should be provided to all men with LUTS, while other advice should only be presented to certain groups. Some recommendations for additions were offered.

**Conclusions:**

Despite a lack of evidence for the self‐management advice, physicians agreed with most of the included advice. The online platform needs further development. Therefore, adjustments will be made and we will assess its impact in future studies.

## 1 INTRODUCTION

The main guidelines for lower urinary tract symptoms (LUTS) in men promote self‐management and lifestyle changes,^1^, ^2^, ^3^ recommending these as the first‐line treatment for patients with mild complaints. This advice is mainly based on the results of one study in which group intervention with self‐management and lifestyle advice led by a urology nurse specialist reduced LUTS severity compared with watchful waiting.^4^ However, it can be time‐consuming and expensive to implement such intervention in daily practice, and more importantly, it requires a hospital setting. Given that patients with LUTS typically present to a general practitioner (GP), self‐management options are perhaps more relevant in primary care. Group sessions also generally provide nonpersonalized information and advice, whereas a more personalized approach could produce additional benefits. Indeed, research has shown that relieving a patient's most bothersome symptom had a greater impact on the quality of life than focusing on the total symptom score in men with LUTS.^5^ Given that online interventions offer a low‐cost option in all care settings, combining a personalized approach with internet technology may provide advantages over group sessions.

To facilitate self‐management and offer lifestyle advice for men with uncomplicated LUTS, we aimed to develop an evidence‐based online intervention that offered advice according to the specific needs and complaints of a given patient.

## 2 MATERIAL AND METHODS

We describe our development of an online platform for the personalized self‐management of uncomplicated LUTS in men. This was based on a scoping review of the relevant literature, usability testing by a small cohort of patients, and a survey of the suitability of the items proposed for inclusion among urologists and GPs. As such this approach reflects the evidence‐based medicine triad, combining scientific evidence with the experiences of physicians and opinions of patients.

### 2.1 Literature review

The scoping review was performed to identify relevant self‐management components for inclusion in the intervention. For this, we searched PubMed from inception to 12th January 2017, using terms related to LUTS, self‐care, and study design (see Supporting Information File 1 for the detailed search strategy). One author (PJ) screened all records (ie, titles and abstracts) to select publications that were potentially relevant or for which the relevance was unclear. Another two authors (PB and MHB) screened the preselected publications and included those focusing on the self‐management of LUTS in men if they were part of a review, guideline or original study. Original studies included randomized controlled trials, uncontrolled trials, and observational studies without specific interventions. The reference lists of the included papers were screened for additional relevant articles by one author (PB).

After identifying relevant components (self‐help advice), we expanded the literature search to find additional evidence for each component. Details of the study design, outcomes, outcome timings, and guideline names were extracted and presented in an evidence table. Based on the available information, we assigned a level of evidence and grade of recommendation to each component, according to the Oxford levels of evidence.^6^ Finally, we rephrased each identified component as a specific item of self‐help advice.

### 2.2 Building the online self‐management intervention

To ensure that patients only received information and advice relevant to them, the online intervention comprised two parts. In part one, the patient completed obligatory questionnaires about their personal characteristics, lifestyle habits, and LUTS severity (International Prostate Symptoms Score,^7^ and Overactive Bladder Questionnaire^8^). They were also asked to complete a frequency‐volume (FV) chart for 1 day. For part two, algorithms then used this information to select and present relevant self‐management advice for that patient. General information about LUTS was presented in an illustrated video message. Other items of advice were presented as text with illustrations. All information was also available in audio format. Patients were free to choose the intensity of usage of the website. After 6 weeks, participants were asked to complete a questionnaire including the same items as the baseline questionnaire, and add information from a new FV chart, to update the personalized advices. The website did not incorporate the presentation of clinical response rates.

### 2.3 Usability testing among men with LUTS

Researchers tested the functionality of the website, by means of entering various patient profiles and checking if algorithms were correct and pieces of advice were applicable to the individual patient (profile). Once satisfied, invited men with uncomplicated LUTS for end‐user testing. Participants were identified from among those referred to a urology outpatient department. They were given access to the intervention and were asked to participate in a semistructured and audio‐recorded face‐to‐face interview after 1 week. One of the authors (BS) performed all interviews at a location of the participant's choosing. Interview topics included the usability, comprehensibility, and design of the website.

### 2.4 Physician survey

When developing and testing the online platform, we also performed an online survey of GPs and urologists. The survey presented the physicians with each advice item and a summary of the available evidence, the level of that evidence, and the grade of the recommendation. Respondents could view the full evidence table by clicking on a link. Two questions were then asked for each item. First, we asked, “Should this advice be included in a self‐management intervention for male patients with uncomplicated LUTS?.” This question was graded on a five‐point Likert scale ranging from “definitively no” to “definitively yes,” with an additional option of “no opinion.” For those who responded “neutral,” “yes” or “definitively yes,” we also asked “Should this advice be given to every patient?,” for which we allowed responses of “yes (to all patients)” or “no (only to specific subgroups).” If a specific subgroup was recommended, they were asked to state the people for whom it should be used. The survey ended with a free‐response question asking if the respondent thought any advice was missing or should be added.

We invited GPs and urologists (including trainees) to complete the survey because these groups most commonly treat LUTS in men. The Dutch Urological Association sent an e‐mail invitation on our behalf to every urologist and urologist in training in the Netherlands, asking them to complete the survey. GPs and GPs in training were invited by e‐mail from among established contacts of the research group. The invitations contained written information about the survey, an option to watch a short movie explaining the background and importance of the survey, a link to the survey, and details of an incentive (€25) for completing the questionnaire. After 2 weeks, reminders were sent using the same distribution channels.

### 2.5 Analysis

The survey results are summarized and presented as frequencies and percentages. Using 5‐point Likert scales, we viewed the answers “definitively yes” and “yes” as positive and “definitively no” and “no” as negative. The in‐training groups were combined with their respective professions for our analyses. However, we refrained from performing inferential statistics because our aim was not to study possible differences between professionals, but rather to identify preferred advice items through consensus. The consensus was considered to be reached when >50% of physicians provided a positive answer (“definitively yes” or “yes”) to the question “Should this advice be included in a self‐management intervention for male patients with uncomplicated LUTS?.”

## 3 RESULTS

### 3.1 Literature review

We identified 828 publications in the primary search, from among which 48 were reviewed in detail. Review of this literature uncovered nine advice items that were applicable to the self‐management of LUTS. These were as follows: (a) information and education about LUTS, (b) pelvic floor muscle training, (c) bladder training, (d) urethral milking, (e) double voiding, (f) caffeine management, (g) alcohol management, (h) fluid management, and (i) exercise advice.

Additional searches to identify further evidence for each advice yielded 31 results (25 reviews or original studies and 6 guidelines). The evidence base for each advice is presented in Table 1, and full details are presented in Supporting Information File 2. We only identified five randomized controlled trials that covered four of the advice items. Most of the evidence originated from studies in which the advice was included in a combined intervention. Furthermore, we identified nine relevant guidelines for male LUTS that each listed between one and seven of the advice recommendations (Table 2).^1^, ^2^, ^3^, ^9^, ^10^, ^11^, ^12^, ^13^, ^14^, ^15^

**Table 1:** Summary of the evidence for advice items in the self‐management intervention

Advice items	RCT	Observational studies^a^	Combined intervention	Number of guidelines	LoE	GoR
1. Information and education	1	1	2	7	2	B
2. Pelvic floor muscle training	2	0	6	5	2	B
3. Bladder training	0	1	6	6	–	–
4. Urethral milking	1	0	2	4	2	B
5. Caffeine management	1	3	4	6	2	B
6. Alcohol management	0	4	3	5	–	–
7. Exercise and weight reduction	0	4	0	4	–	–
8. Fluid management	0	1	5	7	–	–
9. Double voiding	0	0	2	3	–	–

The details are presented in full in Supporting Information File 2.

Abbreviations: GoR, grade of recommendation; LoE, level of evidence; RCT, randomized controlled trial.

^a^ Observational studies included direct comparison studies (non‐RCTs).

**Table 2:** Summary of the advice included in relevant guidelines

	Advice item									
Guideline	1	2	3	4	5	6	7	8	9	Total
European Association of Urology guidance on nonneurogenic male LUTS (2015)^1^	+		+	+	+	+		+	+	7
American Urology Association/Society of Urodynamics, Female Pelvic Medicine and Urogenital Reconstruction, “Non‐Neurogenic Overactive Bladder in Adults” (2012)^9^	+	+	+		+		+	+		6
American Urology Association “Update on AUA guideline on the management of benign prostatic hyperplasia” (2014)^2^	+				+	+		+	+	5
Dutch college of general practitioners’ guideline on Male LUTS (2013)^10^, ^11^	+	+	+	+			+	+		6
Urological Association of Asia “UAA Consensus on the Management of BPH/Male LUTS” (1st Edition) (2012)^12^	+		+	+	+	+	+	+	+	8
6th International Consultation on New Developments in Prostate Cancer and Prostate Diseases (2009)^13^	+	+	+							3
Dutch guideline for physiotherapy in patients with stress urinary incontinence: an update (2014)^14^		+								1
The Japanese Urological Association Clinical guideline for Nocturia (2010)^30^					+	+	+	+		4
NICE guidance on the management of LUTS in men (2015)^3^, ^15^	+	+	+	+	+	+		+		7

Advice numbers refer to the following: 1. Information and education; 2. Pelvic floor muscle training; 3. Bladder training; 4. Urethral milking; 5. Caffeine management; 6. Alcohol management; 7. Exercise and weight reduction; 8. Fluid management; 9. Double voiding. “+” symbol indicates that the advice was included in the guideline.

### 3.2 Results of usability testing among men with LUTS

We invited 42 men to participate in the testing phase. Nine of these tested the intervention and we reached saturation after six interviews. The men reported that they had difficulties completing and understanding the FV chart online and that they would like to receive advice even when the FV chart was not completed. One man wanted more information to be included about prostate cancer. None of the men used the audio files.

### 3.3 Results of the physician survey

**Figure 1:**
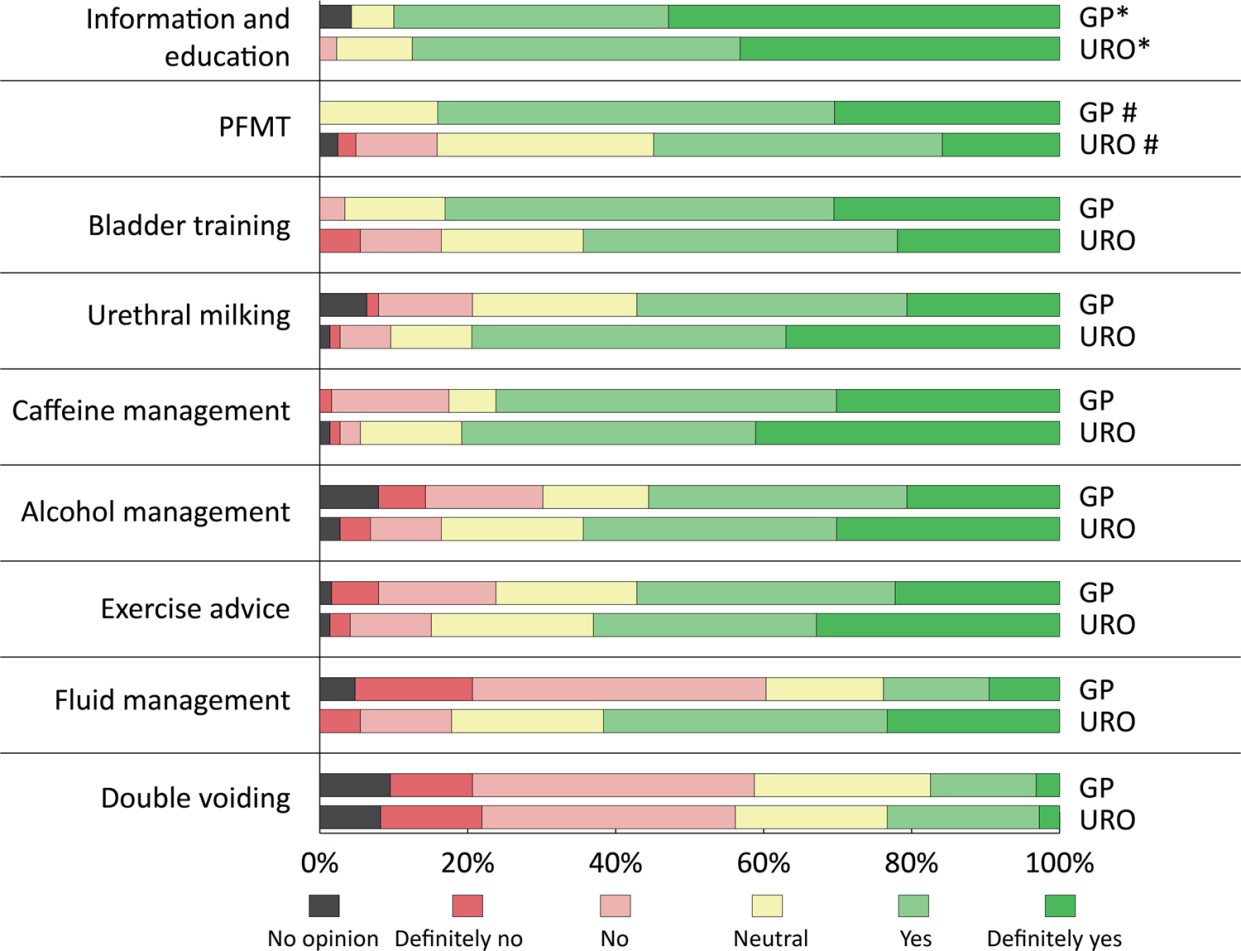
Physician responses to whether each advice item should be included in the self‐management intervention. Data from 70 GPs and 88 urologists were included for the *information and education* advice, from 69 GPs and 82 urologists for the *PFMT* advice, and from 63 GPs and 73 urologists for all other items. GP, general practitioner (including trainees); PFMT, pelvic floor muscle therapy; URO, urologist (including trainees)

E‐mail invitations were sent to 514 physicians in the urologist group, but the number invited in the GP group is unknown. In total, 158 physicians returned the questionnaires (51 GPs, 19 GPs in training, 67 urologists, and 21 urologists in training). In 22 of these cases, one or more answers were missing. The opinions about the inclusion of each advice item are summarized in Figure 1. The consensus was found for seven of the nine advice items.

The highest overall consensus was shown for the item concerning information and education, with most participants answering either “yes” (41%) or “definitively yes” (48%). Of these, 85% judged that the advice should be presented to every patient with LUTS. Equal numbers of physicians advocated bladder training for all patients and specifically for patients with storage problems and frequent micturition. Respectively, 78%, 84%, and 74% of participants advocating advice on caffeine use, alcohol consumption, and exercise felt they should be presented to all patients.

There were some differences between the GP and urologist groups. For example, pelvic floor muscle training was preferred much more by GPs (84%) than by urologists (55%). Among all the physicians advocating the inclusion of pelvic floor muscle training, 62% felt that it should be provided to all patients with LUTS. The others felt it should only be given to patient groups with postvoid dribbling and incontinence. By contrast, urologists (80%) favored urethral milking advice more than GPs (57%), with a slight majority of physicians (55%) feeling this should only be offered to men with postvoid dribbling. Also, although only 43% of all physicians accepted fluid management, its inclusion was disproportionately preferred by urologists (62%) compared with GPs (25%). Finally, all physicians rejected the inclusion of double voiding (80% gave a negative or neutral score).

Forty‐six respondents (34%) felt that some key advice was missing. The most frequently mentioned were advice to use a sitting position when urinating (n = 18), advice related to fluid management (n = 7), and additional detail for the information and education advice (n = 6). Comments about fluid management included the timing of fluid intake in specific patient groups, such as increased fluid intake and nocturia.

## 4 DISCUSSION

We identified nine advice items by literature review and included all in a purpose‐built online platform designed to offer personalized self‐management interventions for men with uncomplicated LUTS. The platform is at an early stage of development. The evidence level was low for much of the included advice, primarily because of the paucity of research in this field. Among the available randomized controlled trials, five had analyzed specific advice,^16^, ^17^, ^18^, ^19^, ^20^ and five had analyzed multicomponent self‐management and lifestyle interventions.^4^, ^21^, ^22^, ^23^, ^24^, ^25^ Consistent with the main guidelines in this field,^1^, ^2^, ^3^, ^9^, ^10^, ^11^, ^12^, ^13^, ^14^, ^15^ the physicians we queried considered most of the advice we included to be relevant for patients and supported its inclusion in the intervention.

The discrepancy between the lack of convincing evidence and the positive recommendations given by physicians may reflect entrenched opinions about specific items of self‐management advice. Indeed, experience in routine daily care may form the basis of these positive recommendations, with the absence of evidence being viewed as very different from the presence of contradictory evidence.^26^ The opinions of physicians may also reflect their wish to involve patients in treatment, viewing lifestyle advice as a harmless means of achieving this goal. Supporting this position, and consistent with their use in many multicomponent intervention studies,^4^, ^21^, ^22^, ^23^, ^24^, ^25^, ^27^ physicians strongly agreed with the inclusion of most of the advice items. In particular, they agreed that general information and education about LUTS should be included for all patients, together with advice about caffeine use, alcohol use, and exercise. However, for the other advice items, around 50% of physicians reported that they should only be given to specific subgroups, promoting personalized care.^5^, ^28^

Despite the consensus to include most items (seven of nine), the need for some alterations were introduced by the physician survey. It was notable, for example, that physicians rejected the advice about double voiding that has been incorporated in three guidelines.^1^, ^2^, ^12^ In our review, we only found evidence to support this advice in two studies that mentioned it as part of a multicomponent intervention.^4^, ^22^ Thus, the lack of evidence and the presence of a consensus to exclude this advice led us to remove it from a more recent iteration of the intervention. Our study protocol required >50% positive responses to include an advice item, which led to the rejection of the fluid management advice (eg, including the neutral scores would have allowed the inclusion of this advice). Notably, there was also a large discrepancy between urologists, who favored the inclusion of this advice (63% “yes” or “definitely yes”), and GPs, who collectively rejected the inclusion of this advice (58% “no” or “definitely no”). This may reflect differences in knowledge about this issue between these groups. We phrased the advice as “to reduce total fluid intake and/or reduce fluid intake at certain times.” Some physicians suggested that some patients may actually benefit from increased fluid intake because concentrated urine may cause LUTS by irritating the bladder. Based on these considerations, we decided to retain this advice, adjusting it slightly to include higher or lower fluid intake as well as the timing of that intake. The advice will be presented when appropriate to the patient's needs.

Many physicians also added the recommendation to include advice to urinate in the sitting position. We only identified one systematic review that evaluated the impact of posture on urodynamic outcomes,^29^ and this revealed no differences in asymptomatic men but beneficial effects for some urodynamic outcomes in men with LUTS. Although the clinical relevance of these outcomes was unclear, this advice will be included in future interventions.

The usability evaluation by patients revealed other changes that needed to be made to our online intervention. Patients encountered problems when completing the online FV chart, but we believe these can be overcome with simple technical adjustments of the website (eg, using clearer instructions). The impact of the nine‐item version of the intervention is currently under study. For future use, we will adjust the intervention to include the following items: information and education about LUTS, pelvic floor muscle training, bladder training, urethral milking, caffeine management, alcohol management, fluid management, exercise and advice to use the sitting position. We believe that, in addition to providing general information and education to all men with uncomplicated LUTS, presenting advice only to patients for whom it is relevant will have a beneficial impact on their quality of life and symptom severity.

Our study has some limitations. First, we did not perform a systematic review of the literature with a risk of bias assessment. We restricted ourselves to descriptions of the study type and characteristics to provide an overview of the limited evidence available on this topic. Second, the unstructured invitation process for GPs means that we do not know the response rate in this group, although we know that the response rate was low for the urologist group (17%). Thus, we do not know whether our sample is representative of these physician groups, but we assume that those with a special interest in functional urology are overrepresented. Moreover, the study population was probably too small to allow generalization. Third, the consensus among physicians was defined at an arbitrary threshold, and as discussed, a less strict criterion may have been more appropriate. Fourth, in this pilot study, we did not apply standardized questionnaires that allow the evaluation of medical apps and websites. In our future more large‐scale studies we will apply such questionnaires.

Despite a lack of convincing evidence for the included advice, most urologists and GPs agreed with the use of seven of nine proposed items in an online self‐management intervention, further suggesting the inclusion of another item. It was also evident that some elements should be made available for all men with LUTS, whereas other elements may need to be offered based on symptom type, symptom severity, bladder diaries, and other features.

We have evaluated the effect of applying the nine‐item version of the platform in men who were referred to the urology outpatient department of three hospitals. Details of that study are published elsewhere (NAU‐19‐0154). We will further iterate the intervention based on the outcomes of the physicians survey, and additionally study patient expectations, and patient experiences in the primary care setting. If we demonstrate the added value of this online intervention, we will make it available for patients.

## CONFLICT OF INTERESTS

The authors declare that they have no conflict of interests.

## Supporting information

Supporting information

Supporting information

Supporting information
