# Supplementary material for: Development of an online personalized self‐management intervention for men with uncomplicated LUTS
Source: Neurourol Urodyn. 2019 May 20;38(6):1685–91. doi: 10.1002/nau.24040 (PMC6851544; doi:10.1002/nau.24040)
Supplement: Supplementary file 1 — Supporting information [file NAU-38-1685-s001.docx]

**Supplementary file 1 Search strategy**

The search strategy was built, based upon other published reviews, and relevant papers, using the PICO system.

**P**ICOs: LUTS

P**I**COs: self-management

PICO**S**: publication types or guidelines

Exclusion: studies in women only and animal studies

For **LUTS** we used the following terms:

(lower urinary tract symptoms[MESH] OR lower urinary tract symptoms[tiab] OR LUTS[tiab] OR urinary symptom*[tiab] OR dysuria[tiab] OR nocturia[tiab] OR prostatism[tiab] OR (overactiv*[tiab] AND (bladder[tiab] OR detrusor[tiab])) OR urinary incontinence[MESH] OR (urinary[tiab] AND incontinence[tiab]) OR prostatic hyperplasia[MESH] OR ((prostate[tiab] OR prostatic[tiab]) AND (hyperplasia[tiab] OR hypertrophy[tiab] OR BPH[tiab] OR enlargement[tiab] OR obstruction[tiab] OR adenoma*[tiab])) OR urination disorders[MESH] OR urination disorder*[tiab] OR urinary disorder*[tiab] OR polyuria[tiab] OR voiding dysfunction[tiab] OR voiding disorder*[tiab] OR urinary bladder neck obstruction[MESH] OR bladder neck obstruction[tiab] OR bladder outlet obstruction[tiab] OR ((urine[tiab] OR urinary[tiab]) AND (residual[tiab] OR retention[tiab])) OR incomplete voiding[tiab])

For s**elf-management** we used the following terms:

(self care[MESH] OR self-care[tiab] OR self care[tiab] OR self-management[tiab] OR self management[tiab] OR self efficacy[MESH] OR self-efficacy[tiab] OR self efficacy[tiab] OR patient education as topic[MESH] OR patient education[tiab] OR consumer health information[MESH] OR health education[MESH] OR health education[tiab] OR educational intervention*[tiab] OR educational material*[tiab] OR patient empowerment[tiab] OR patient engagement[tiab] OR patient activation[tiab] OR patient involvement[tiab] OR patient cent*[tiab] OR patient-cent*[tiab] OR ((problem solving[tiab] OR problem-solving[tiab] OR goal setting[tiab] OR goal-setting[tiab]) AND skill*[tiab]) OR lifestyle modification*[tiab] OR lifestyle intervention*[tiab] OR behavior chang*[tiab] OR behaviour chang*[tiab] OR behavioral chang*[tiab] OR behavioural chang*[tiab] OR behavior intervention*[tiab] OR behaviour intervention*[tiab] OR behavioral intervention*[tiab] OR behavioural intervention*[tiab] OR action plan*[tiab])

For **publication types** we applied the *Cochrane Highly Sensitive Search Strategy for identifying randomized trials*, and used terms for systematic reviews, and consensus methods:

(randomized controlled trial[pt] OR controlled clinical trial[pt] OR randomized[tiab] OR randomised[tiab] OR placebo[tiab] OR clinical trials as topic[mesh:noexp] OR randomly[tiab] OR trial[ti] OR Review[pt] OR Meta-Analysis[pt] OR Cochrane[tiab] OR systematic review[tiab] OR practice guideline*[tiab] OR Consensus[mesh] OR consensus method*[tiab] OR consensus process[tiab] OR consensus meeting[tiab] OR panel meeting[tiab] OR expert panel[tiab] OR multidisciplinary panel[tiab] OR consultation process[tiab] OR ((apprais*[tiab] OR rate*[tiab] OR defin*[tiab] OR develop*[tiab]) AND (component*[tiab] OR content*[tiab] OR program*[tiab] OR guideline*[tiab])) OR Delphi Technique[mesh] OR Delphi[tiab])

For **guidelines** we used:

(Guideline[pt] OR Practice Guideline[pt])

For **exclusions** we used the following terms:

((animals[mh] NOT humans[mh]) OR ((women[tiab] OR female[tiab]) NOT (men[tiab] OR male[tiab])))

The final search included:

((LUTS AND self-management AND publication types) OR (LUTS AND guidelines)) NOT exclusions.
