# Supplementary material for: Development of an online personalized self‐management intervention for men with uncomplicated LUTS
Source: Neurourol Urodyn. 2019 May 20;38(6):1685–91. doi: 10.1002/nau.24040 (PMC6851544; doi:10.1002/nau.24040)
Supplement: Supplementary file 2 — Supporting information [file NAU-38-1685-s002.docx]

**Supplementary file 2 – detailed description of the evidence base for the nine advices found in the scoping review**

In the survey among physicians, the advice, level of evidence, grade of recommendation and the evidence summary were shown. The additional information presented in the Table was visible if participants selected the option to read more.

Please note that the NICE guideline was not included in the data provided in the survey amongst the physicians, despite the fact that it had served as an important source of information in the primary search. We have added this guideline to the Tables as it should have been presented.

**Advice 1: information**

Information about the normal and abnormal function of the urinary tract. Explaining that LUTS are often innocent and prostate cancer almost never is the cause of LUTS.

**Level of evidence: 2**

**Grade of recommendation: B**

In a Dutch intervention study, the effect of an education program for GPs in the field of LUTS was investigated. This showed that patients who came to a GP who had followed the education program could better deal with their LUTS than patients who came to a GP who had not followed this program.

A British qualitative study in which men with LUTS were interviewed showed that most men had fear of prostate cancer. After this fear was abated, the majority of these men experienced their complaints as less annoying and better to deal with.

In two studies this advice was part of a combined intervention to reduce LUTS (OAB, nocturia, urinary incontinence).

The advice is stated in five guidelines.

| **Intervention studies specifically on this advice** | **Result** |
| --- | --- |
| Wolters et al (2004) ^1^  RCT in which the effect of an intervention program in which the GP was trained (intervention, n = 32 practices, 76 patients) was compared with only the distribution of the current guidelines (control, n = 31 practices, 75 patients).  The outcome measure was the extent to which male patients could cope with urinary problems. | To be able to cope better with urinary problems:  Intervention group: 44%  Control group: 28%  Difference: OR 2.21 (CI95% 1.03 – 4.75) |
| **Qualitative studies on this advice** |  |
| Brown et al (2003) ^2^  Qualitative study in which men with LUTS were interviewed about their complaints and what they thought were the cause of their symptoms, mainly focused on fear of prostate cancer. | 73% of men had fear of prostate cancer in relation to their LUTS. Despite the fact that the symptoms had not or hardly changed, 68% of the men indicated that their complaints were less annoying and better to deal with after being reassured that they had no prostate cancer. |
| **Item is part of an intervention in the following studies** | **Outcomes of total intervention** |
| Brown et al (2007) and Yap et al (2009) ^3, 4^  RCT in which the effect of group sessions, supervised by a nurse and focused on self-management (intervention, n = 73 men), was compared with standard care (control, n = 67) in men with LUTS. The outcome measure was Treatment failure, defined as IPSS increase of 3 or more, use of medication for LUTS, acute urinary retention or surgery. Secondary outcomes were number of voids, nocturia episodes and LUTS severity assessed with the IPSS. | Treatment failure (intervention vs. control)  3 months: 10% vs. 42% *  6 months: 19% vs. 61% *  12 months: 31% vs. 79% *  Number of voids (intervention vs. control)  3 months: 7.1 ± 2.1 vs. 9.7 ± 3.3 *  6 months: 7.3 ± 2.2 vs. 9.0 ± 2.1 *  12 months: 7.4 ± 2.3 vs. 9.0 ± 2.6 *  Episodes of nocturia (intervention vs. control)  3 months: 1.3 ± 0.8 vs. 2.0 ± 1.4 *  6 months: 1.1 ± 0.8 vs. 1.9 ± 0.9 *  12 months: 1.0 ± 0.6 vs. 1.6 ± 1.2 *  LUTS severity assessed with IPSS (intervention vs. control)  3 months: 10.7 ± 5.9 vs. 16.4 ± 5.8 *  6 months: 10.4 ± 6.1 vs. 16.9 ± 6.4 *  12 months: 10.2 ± 6.1 vs. 15.4 ± 6.6 * |
| Chen et al. (2012) ^5^  RCT in which the effect of a self-management intervention (n = 119) was compared to regular care (n = 102) in men with BPH. Intervention was offered by means of 2 hours face to face demonstration and a follow up telephone consultation.  The outcome measure was LUTS severity assessed with the IPSS. | LUTS severity assessed with the IPSS (intervention vs. control)  Week 1: 20.6 vs. 19.4  Month 3: 14.3 ± 5.6 vs. 19.7 ± 6.5 *  Month 6: 12.0 ± 5.3 vs. 19.9 ± 6.4 * |
| **This advice is specifically mentioned in the following guidelines** | |
| - European Association of Urology (EAU) Guidelines ‘Non Neurogenic Male LUTS’ (2015) ^6^ | |
| - American Urology Association (AUA)/Society of Urodynamics, Female Pelvic Medicine and Urogenital Reconstruction (SUFU) guideline ‘OAB (non-neurogenic)’ (2014) ^7^ | |
| - American Urology Association (AUA) ‘BPH guideline’ (2014) ^8^ | |
| - Dutch college of general practitioners guideline Male LUTS (2013) ^9, 10^ | |
| - Urological Association of Asia (UAA) UAA Consensus on the Management of BPH/Male LUTS (1st Edition) (2012) ^11^ | |
| - 6th International Consultation on New Developments in Prostate Cancer and Prostate Diseases (2009) ^12^ | |
| - *NICE Guideline Lower urinary tract symptoms in men: management* ^13, 14^ | |

* Statistically significant, p <0.01

**Advice 2: Training of the pelvic floor muscles**

The goal of pelvic floor muscle training is to reduce urinary incontinence, reduce the frequency of micturition and to reduce post-micturition dribble.

**Level of evidence: 2**
**Grade of recommendation: B**

Two intervention studies have investigated the effect of pelvic floor muscle training on post-micturition dribble. One study in men with erectile dysfunction (n = 5), and one study in men with only post-micturition dribble as a complaint (n = 44). Both studies showed a reduction of post-micturition dribble after pelvic floor muscles training.

In 6 studies, pelvic floor muscle training was part of a combined intervention.

The advice is stated in 4 guidelines.

| **Intervention studies specifically on this advice** | **Outcomes** |
| --- | --- |
| Dorey et al. (2004) ^15^  RCT in which the effect of pelvic floor muscle training in addition to lifestyle advice (intervention, n = 28) is compared with lifestyle advice only (control, n = 27) in men with erectile dysfunction. The outcome measure was percentage of men without post-micturition dribbling after three months. | Percentage of men without post-micturition dribbling after three months.  Intervention group: 66.7%  Control group: 6.7%  Intervention vs. control: p < 0.01. |
| Paterson et al. (1997) ^16^  RCT in which the effects of pelvic floor muscle training (n = 15), urethral milking (n = 15) and counselling (n = 14) are compared in men with the complaint of post-micturition dribbling. The outcome measure was the improvement in pad weight gain. | Adjusted mean improvement in pad weight gain (measured during baseline and after 12 weeks).  PFMT vs. counselling: 4.7 grams  Urethral milking vs. counselling: 2.8 grams |
| **Item is part of an intervention in the following studies** | **Outcomes of total intervention** |
| Borrie et al. (2002) ^17^  RCT in which the effect of counselling by an incontinence consultant (intervention, n = 210 of which 68 men) is compared with no care (control, n = 211 of which 66 men) in patients with incontinence. The outcome measure was the mean change in number of incontinence episodes per 24 hours , and change of incontinence material use in pieces. | Mean change in number of incontinence episodes per 24 hours  Intervention group: -1.2  Control group: -0.2  Intervention vs. control: p < 0.01.  Change of incontinence material use in pieces  Intervention group: -0.9  Control group: +0.1  Intervention vs. control: p < 0.01. |
| Brown et al (2007) and Yap et al (2009) ^3, 4^  RCT in which the effect of group sessions, supervised by a nurse and focused on self-management (intervention, n = 73 men), was compared with standard care (control, n = 67) in men with LUTS. The outcome measure was Treatment failure, defined as IPSS increase of 3 or more, use of medication for LUTS, acute urinary retention or surgery. Secondary outcomes were number of voids, nocturia episodes and LUTS severity assessed with the IPSS. | Treatment failure (intervention vs. control)  3 months: 10% vs. 42% *  6 months: 19% vs. 61% *  12 months: 31% vs. 79% *  Number of voids (intervention vs. control)  3 months: 7.1 ± 2.1 vs. 9.7 ± 3.3 *  6 months: 7.3 ± 2.2 vs. 9.0 ± 2.1 *  12 months: 7.4 ± 2.3 vs. 9.0 ± 2.6 *  Episodes of nocturia (intervention vs. control)  3 months: 1.3 ± 0.8 vs. 2.0 ± 1.4 *  6 months: 1.1 ± 0.8 vs. 1.9 ± 0.9 *  12 months: 1.0 ± 0.6 vs. 1.6 ± 1.2 *  LUTS severity assessed with IPSS (intervention vs. control)  3 months: 10.7 ± 5.9 vs. 16.4 ± 5.8 *  6 months: 10.4 ± 6.1 vs. 16.9 ± 6.4 *  12 months: 10.2 ± 6.1 vs. 15.4 ± 6.6 * |
| Chen et al. (2012) ^5^  RCT in which the effect of a self-management intervention (n = 119) was compared to regular care (n = 102) in men with BPH. Intervention was offered by means of 2 hours face to face demonstration and a follow up telephone consultation.  The outcome measure was LUTS severity assessed with the IPSS. | LUTS severity assessed with the IPSS (intervention vs. control)  Week 1: 20.6 vs. 19.4  Month 3: 14.3 ± 5.6 vs. 19.7 ± 6.5 *  Month 6: 12.0 ± 5.3 vs. 19.9 ± 6.4 * |
| Ruiz et al. (2011) ^18^  Intervention study without a control group in which the effect of an e-health website was measured in men and women with OAB (n = 25 of which 13 were men). The outcome measure was the change in LUTS assessed with the OAB questionnaire. | LUTS assessed with the OAB questionnaire at baseline and after 6 weeks of use of the intervention  Scores in men: pre 49.8 ± 21.1 vs. post 29.8 ± 13.9 * |
| Burgio et al. (2011) ^19^  RCT in which the effect of a behavioural treatment and α-blockers (intervention, n = 73) was compared with oxybutynin + α-blockers (n = 70) in men with OAB. The outcome measures were change in 24-hour voiding frequency and nocturnal frequency, and change in LUTS severity assessed with AUA symptom index after 8 weeks. | Change in 24-hour voiding frequency after 8 weeks.  Intervention group: 11.3 ± 2.5 to 9.1 ± 2.5  Control group: 11.5 ± 2.1 to 9.5 ± 2.4  Equivalence test: p <0.01, this means the same treatment effect in both groups.  Change in nocturnal voiding frequency after 8 weeks.  Intervention group: -0.70 ± 0.72  Control group: -0.32 ± 1.28  Intervention vs. control: p<0.01  Change in LUTS severity assessed with AUA symptom index after 8 weeks.  Intervention group: -3.4 ± 4.6  Control group: -3.2 ± 5.6  Intervention vs. control: not statistically significant |
| Johnson et al. (2016) ^20^  RCT in which the effect of a behavioural treatment to reduce nocturia (behaviour group, n = 23) was compared with behavioural treatment with α-blockers (combination group, n = 25) and α-blockers alone (α-blocker group, n = 24) in men with nocturia. Outcome measure was change in number of nocturia episodes after 12 weeks. | Change in number of nocturia episodes after 12 weeks, compared to baseline.  Behavioural group: -1.39 ± 1.5  Combination group: -1.03 ± 1.4  α-blocker group: -0.59 ± 0.9  Difference between the groups: not statistically significant |
| **This advice is specifically mentioned in the following guidelines** | |
| - American Urology Association (AUA)/Society of Urodynamics, Female Pelvic Medicine and Urogenital Reconstruction (SUFU) guideline ‘OAB (non-neurogenic)’ (2014) ^7^ | |
| - Dutch college of general practitioners guideline Male LUTS (2013) ^9, 10^ | |
| - Dutch guideline for physiotherapy in patients with stress urinary incontinence: an update (2014) ^21^ | |
| - 6th International Consultation on New Developments in Prostate Cancer and Prostate Diseases (2009) ^12^ | |
| - *NICE Guideline Lower urinary tract symptoms in men: management* ^13, 14^ | |

* Statistically significant, p <0.01

**Advice 3: bladder training**

The purpose of bladder training is to increase the time between micturitions by means of postpone the micturitions at urgency.

**Level of evidence: -**

**Grade of recommendation: -**

In one intervention study, the effect of bladder training combined with tolterodine versus tolterodine alone was studied in patients with OAB (n = 501, 75% female). Both groups had an improvement after 24 weeks in the number of micturitions, incontinence episodes and urgency episodes per day. The voiding frequency decreased more with bladder training than with tolterodine alone. In incontinence and urgency episodes, there was no difference between the groups.

In 6 studies the advice is part of a combined intervention.

The advice is stated in 5 guidelines.

| **Intervention studies specifically on this advice** | **Outcomes** |
| --- | --- |
| Mattiasson et al. (2003) ^22^  RCT in which the effect of bladder training and tolterodine (intervention, n = 244 of which 67 men) was compared with tolterodine alone (control, n = 257 of which 56 men) in patients with OAB. The outcome measures were the number of daily micturitions, incontinence episodes per day, and urgency episodes, and in mean voided volume after 24 weeks | Both groups had a reduction in the number of daily micturitions, incontinence episodes per day, and urgency episodes, and increase in mean voided volume after 24 weeks  However, only the 24 hour voiding frequency decreased more strongly in the intervention group than the control group (percentage improvement (median) 33% vs. 25%)*. |
| **Item is part of an intervention in the following studies** | **Outcomes of total intervention** |
| Borrie et al. (2002) ^17^  RCT in which the effect of counselling by an incontinence consultant (intervention, n = 210 of which 68 men) is compared with no care (control, n = 211 of which 66 men) in patients with incontinence. The outcome measure was the mean change in number of incontinence episodes per 24 hours , and change of incontinence material use in pieces. | Mean change in number of incontinence episodes per 24 hours  Intervention group: -1.2  Control group: -0.2  Intervention vs. control: p < 0.01.  Change of incontinence material use in pieces  Intervention group: -0.9  Control group: +0.1  Intervention vs. control: p < 0.01. |
| Brown et al (2007) and Yap et al (2009) ^3, 4^  RCT in which the effect of group sessions, supervised by a nurse and focused on self-management (intervention, n = 73 men), was compared with standard care (control, n = 67) in men with LUTS. The outcome measure was Treatment failure, defined as IPSS increase of 3 or more, use of medication for LUTS, acute urinary retention or surgery. Secondary outcomes were number of voids, nocturia episodes and LUTS severity assessed with the IPSS. | Treatment failure (intervention vs. control)  3 months: 10% vs. 42% *  6 months: 19% vs. 61% *  12 months: 31% vs. 79% *  Number of voids (intervention vs. control)  3 months: 7.1 ± 2.1 vs. 9.7 ± 3.3 *  6 months: 7.3 ± 2.2 vs. 9.0 ± 2.1 *  12 months: 7.4 ± 2.3 vs. 9.0 ± 2.6 *  Episodes of nocturia (intervention vs. control)  3 months: 1.3 ± 0.8 vs. 2.0 ± 1.4 *  6 months: 1.1 ± 0.8 vs. 1.9 ± 0.9 *  12 months: 1.0 ± 0.6 vs. 1.6 ± 1.2 *  LUTS severity assessed with IPSS (intervention vs. control)  3 months: 10.7 ± 5.9 vs. 16.4 ± 5.8 *  6 months: 10.4 ± 6.1 vs. 16.9 ± 6.4 *  12 months: 10.2 ± 6.1 vs. 15.4 ± 6.6 * |
| Chen et al. (2012) ^5^  RCT in which the effect of a self-management intervention (n = 119) was compared to regular care (n = 102) in men with BPH. Intervention was offered by means of 2 hours face to face demonstration and a follow up telephone consultation.  The outcome measure was LUTS severity assessed with the IPSS. | LUTS severity assessed with the IPSS (intervention vs. control)  Week 1: 20.6 vs. 19.4  Month 3: 14.3 ± 5.6 vs. 19.7 ± 6.5 *  Month 6: 12.0 ± 5.3 vs. 19.9 ± 6.4 * |
| Ruiz et al. (2011) ^18^  Intervention study without a control group in which the effect of an e-health website was measured in men and women with OAB (n = 25 of which 13 were men). The outcome measure was the change in LUTS assessed with the OAB questionnaire. | LUTS assessed with the OAB questionnaire at baseline and after 6 weeks of use of the intervention  Scores in men: pre 49.8 ± 21.1 vs. post 29.8 ± 13.9 * |
| Burgio et al. (2011) ^19^  RCT in which the effect of a behavioural treatment and α-blockers (intervention, n = 73) was compared with oxybutynin + α-blockers (n = 70) in men with OAB. The outcome measures were change in 24-hour voiding frequency and nocturnal frequency, and change in LUTS severity assessed with AUA symptom index after 8 weeks. | Change in 24-hour voiding frequency after 8 weeks.  Intervention group: 11.3 ± 2.5 to 9.1 ± 2.5  Control group: 11.5 ± 2.1 to 9.5 ± 2.4  Equivalence test: p <0.01, this means the same treatment effect in both groups.  Change in nocturnal voiding frequency after 8 weeks.  Intervention group: -0.70 ± 0.72  Control group: -0.32 ± 1.28  Intervention vs. control: p<0.01  Change in LUTS severity assessed with AUA symptom index after 8 weeks.  Intervention group: -3.4 ± 4.6  Control group: -3.2 ± 5.6  Intervention vs. control: not statistically significant |
| Johnson et al. (2016) ^20^  RCT in which the effect of a behavioural treatment to reduce nocturia (behaviour group, n = 23) was compared with behavioural treatment with α-blockers (combination group, n = 25) and α-blockers alone (α-blocker group, n = 24) in men with nocturia. Outcome measure was change in number of nocturia episodes after 12 weeks. | Change in number of nocturia episodes after 12 weeks, compared to baseline.  Behavioural group: -1.39 ± 1.5  Combination group: -1.03 ± 1.4  α-blocker group: -0.59 ± 0.9  Difference between the groups: not statistically significant |
| **This advice is specifically mentioned in the following guidelines** | |
| - European Association of Urology (EAU) Guidelines ‘Non Neurogenic Male LUTS’ (2015) ^6^ | |
| - American Urology Association (AUA)/Society of Urodynamics, Female Pelvic Medicine and Urogenital Reconstruction (SUFU) guideline ‘OAB (non-neurogenic)’ (2014) ^7^ | |
| - Dutch college of general practitioners guideline Male LUTS (2013) ^9, 10^ | |
| - Urological Association of Asia (UAA) UAA Consensus on the Management of BPH/Male LUTS (1st Edition) (2012) ^11^ | |
| - 6th International Consultation on New Developments in Prostate Cancer and Prostate Diseases (2009) ^12^ | |
| - *NICE Guideline Lower urinary tract symptoms in men: management* ^13, 14^ | |

* Statistically significant, p <0.01

**Advice 4: Emptying urethra (urethral milking)**

The purpose of this is to reduce or prevent post-micturition dribbling.

**Level of evidence: 2**

**Grade of recommendation: B**

In one intervention study, the effect of emptying the urethra on the reduction of post-micturition dribbling was investigated, whereby a reduction of fluid collected in bandaging material was measured.

In 2 studies the advice was part of a combined intervention.

The advice is stated in 3 guidelines.

| **Intervention studies specifically on this advice** | **Outcomes** |
| --- | --- |
| Paterson et al. (1997) ^16^  RCT in which the effects of pelvic floor muscle training (n = 15), urethral milking (n = 15) and counselling (n = 14) are compared in men with the complaint of post-micturition dribbling. The outcome measure was the improvement in pad weight gain. | Adjusted mean improvement in pad weight gain (measured during baseline and after 12 weeks).  PFMT vs. counselling: 4.7 grams  Urethral milking vs. counselling: 2.8 grams |
| **Item is part of an intervention in the following studies** | **Outcomes of total intervention** |
| Brown et al (2007) and Yap et al (2009) ^3, 4^  RCT in which the effect of group sessions, supervised by a nurse and focused on self-management (intervention, n = 73 men), was compared with standard care (control, n = 67) in men with LUTS. The outcome measure was Treatment failure, defined as IPSS increase of 3 or more, use of medication for LUTS, acute urinary retention or surgery. Secondary outcomes were number of voids, nocturia episodes and LUTS severity assessed with the IPSS. | Treatment failure (intervention vs. control)  3 months: 10% vs. 42% *  6 months: 19% vs. 61% *  12 months: 31% vs. 79% *  Number of voids (intervention vs. control)  3 months: 7.1 ± 2.1 vs. 9.7 ± 3.3 *  6 months: 7.3 ± 2.2 vs. 9.0 ± 2.1 *  12 months: 7.4 ± 2.3 vs. 9.0 ± 2.6 *  Episodes of nocturia (intervention vs. control)  3 months: 1.3 ± 0.8 vs. 2.0 ± 1.4 *  6 months: 1.1 ± 0.8 vs. 1.9 ± 0.9 *  12 months: 1.0 ± 0.6 vs. 1.6 ± 1.2 *  LUTS severity assessed with IPSS (intervention vs. control)  3 months: 10.7 ± 5.9 vs. 16.4 ± 5.8 *  6 months: 10.4 ± 6.1 vs. 16.9 ± 6.4 *  12 months: 10.2 ± 6.1 vs. 15.4 ± 6.6 * |
| Chen et al. (2012) ^5^  RCT in which the effect of a self-management intervention (n = 119) was compared to regular care (n = 102) in men with BPH. Intervention was offered by means of 2 hours face to face demonstration and a follow up telephone consultation.  The outcome measure was LUTS severity assessed with the IPSS. | LUTS severity assessed with the IPSS (intervention vs. control)  Week 1: 20.6 vs. 19.4  Month 3: 14.3 ± 5.6 vs. 19.7 ± 6.5 *  Month 6: 12.0 ± 5.3 vs. 19.9 ± 6.4 * |
| **This advice is specifically mentioned in the following guidelines** | |
| - European Association of Urology (EAU) Guidelines ‘Non Neurogenic Male LUTS’ (2015) ^6^ | |
| - Dutch college of general practitioners guideline Male LUTS (2013) ^9, 10^ | |
| - Urological Association of Asia (UAA) UAA Consensus on the Management of BPH/Male LUTS (1st Edition) (2012) ^11^ | |
| - *NICE Guideline Lower urinary tract symptoms in men: management* ^13, 14^ | |

* Statistically significant, p <0.01

**Advice 5: double voiding**

Advice to wait a little while after the first urination, lean forward slightly and then urinate again.

**Level of evidence: -**

**Grade of recommendation: -**

There are no intervention studies that have investigated the effect of double voiding.

In 2 studies this advice was part of a combined intervention.

It is mentioned in 3 guidelines.

| **Intervention studies specifically on this advice** |  |
| --- | --- |
| None |  |
| **Item is part of an intervention in the following studies** | **Outcomes of total intervention** |
| Brown et al (2007) and Yap et al (2009) ^3, 4^  RCT in which the effect of group sessions, supervised by a nurse and focused on self-management (intervention, n = 73 men), was compared with standard care (control, n = 67) in men with LUTS. The outcome measure was Treatment failure, defined as IPSS increase of 3 or more, use of medication for LUTS, acute urinary retention or surgery. Secondary outcomes were number of voids, nocturia episodes and LUTS severity assessed with the IPSS. | Treatment failure (intervention vs. control)  3 months: 10% vs. 42% *  6 months: 19% vs. 61% *  12 months: 31% vs. 79% *  Number of voids (intervention vs. control)  3 months: 7.1 ± 2.1 vs. 9.7 ± 3.3 *  6 months: 7.3 ± 2.2 vs. 9.0 ± 2.1 *  12 months: 7.4 ± 2.3 vs. 9.0 ± 2.6 *  Episodes of nocturia (intervention vs. control)  3 months: 1.3 ± 0.8 vs. 2.0 ± 1.4 *  6 months: 1.1 ± 0.8 vs. 1.9 ± 0.9 *  12 months: 1.0 ± 0.6 vs. 1.6 ± 1.2 *  LUTS severity assessed with IPSS (intervention vs. control)  3 months: 10.7 ± 5.9 vs. 16.4 ± 5.8 *  6 months: 10.4 ± 6.1 vs. 16.9 ± 6.4 *  12 months: 10.2 ± 6.1 vs. 15.4 ± 6.6 * |
| Chen et al. (2012) ^5^  RCT in which the effect of a self-management intervention (n = 119) was compared to regular care (n = 102) in men with BPH. Intervention was offered by means of 2 hours face to face demonstration and a follow up telephone consultation.  The outcome measure was LUTS severity assessed with the IPSS. | LUTS severity assessed with the IPSS (intervention vs. control)  Week 1: 20.6 vs. 19.4  Month 3: 14.3 ± 5.6 vs. 19.7 ± 6.5 *  Month 6: 12.0 ± 5.3 vs. 19.9 ± 6.4 * |
| **This advice is specifically mentioned in the following guidelines** | |
| - European Association of Urology (EAU) Guidelines ‘Non Neurogenic Male LUTS’ (2015) ^6^ | |
| - American Urology Association (AUA)/Society of Urodynamics, Female Pelvic Medicine and Urogenital Reconstruction (SUFU) guideline ‘OAB (non-neurogenic)’ (2014) ^7^ | |
| - Urological Association of Asia (UAA) UAA Consensus on the Management of BPH/Male LUTS (1st Edition) (2012) ^11^ | |

* Statistically significant, p <0.01

**Advice 6: caffeine use**

Explanation about the possible effect of caffeine on the bladder function. Reduce caffeine use.

**Level of evidence: 2**

**Grade of recommendation: B**

In one intervention study, the effect of caffeine reduction in patients with micturition symptoms (n = 95, 90% woman) was studied. This showed that a reduction to less than 100 mg of caffeine per day resulted in a reduction in the number of micturitions per day and the number of urgency events compared to a control group that drank an average of 240 mg of caffeine. The population of this study consisted mainly of women.

The effect of caffeine on the urinary tract has been investigated in a number of observational studies. For example, one study showed that the diuresis increased 1 hour after caffeine intake, and another study showed that the total volume of urinating was higher after caffeine intake than without caffeine intake. This study also showed that after caffeine intake the urgency to void already occurred with a lower bladder volume.

A large prospective cohort study (n = 1,101) showed that drinking more than 2 cups of coffee per day doubled the likelihood of worsening LUTS.

In 4 studies, this advice was part of a combined intervention.

The advice is stated in 4 guidelines.

| **Intervention studies specifically on this advice** | **Outcomes** |
| --- | --- |
| Bryant et al. (2002) ^23^  RCT in which the effect of caffeine reduction and bladder training (n = 48) was compared with bladder training alone (n = 47), in patients with micturition problems. 90% of the patients were women. The outcome measures were caffeine intake, 24-hour voiding frequency and number of urgency episodes. | Caffeine intake  Intervention group: from 238.7 ± 121 to 96.5 ± 101.8 (58% reduction)  Control group: from 272 ± 184 to 240.5 ± 152.8 (11% reduction)  Intervention vs. control: p<0.01  24-hour voiding frequency  Intervention group: from 11.4 ± 4.0 to 6.8 ± 2.0 (35% reduction)  Control group: from 11.2 ± 3.5 to 7.9 ± 2.6 (23% reduction)  Intervention vs. control: p<0.05  Number of urgency episodes  Intervention group: from 4.8 ± 3.5 to 1.6 ± 1.9 (61% reduction)  Control group: from 4.6 ± 3.0 to 3.2 ± 2.8 (12% reduction)  Intervention vs. control: p<0.01 |
| **Intervention studies effect of caffeine on the urinary tract** |  |
| Nussberger et al. (1990) ^24^  Crossover study of the effect of 300 ml of coffee containing 250 g of caffeine (intervention) compared to 300 ml of decaffeinated coffee (control) on diuresis in healthy men (n = 8). There was at least one week between the two measurements. The outcome measure was change in diuresis. | Change in diuresis  Intervention: from 215 ± 60 ml / hour (for caffeine intake) to 362 ± 48 ml / hour (1 hour after caffeine intake)  Control: none.  Intervention vs. control: p<0.01 |
| Lohsiriwat et al. (2010) ^25^  Crossover study of the effect of caffeine (intervention) compared with water (control) on bladder function in patients with OAB symptoms. During the intervention, patients received 4.5 mg / kg body weight of caffeine dissolved in 8 ml / kg body weight of water. During the control, patients were given 8 ml / kg body weight of water. | Intake of caffeine caused an increase in voided volumes compared to the intake of water alone. Caffeine intake also ensured that the first urgency to void and normal urinary urgency occurred with a significantly lower bladder volume. Data were only presented as figures, no numbers are presented. |
| **Observational studies on this advice** |  |
| Maserejian et al. (2013) ^26^  Observational longitudinal study in which, among other things, the effect of consumption of caffeine and carbonated drinks on progression of LUTS was investigated. The outcome was risk of LUTS progression. | Risk of LUTS progression more likely if   - More than 2 cups of coffee / day (adjusted OR 2.09, 95% CI 1.29-3.40) - More than 1 glass / day of non-sugar-free cola (adjusted OR 1.75, 95% CI 1.04-2.96)   More than 2 cups / day of coffee or caffeine tea in the week before follow up was associated with worsening LUTS.  Men with more than 2 drinks / day of caffeine-containing beverages in the week before follow-up more frequently reported urgency symptoms (adjusted OR 2.06, 95% CI 1.23-3.43).  An increase in consumption of 2 or more cups of coffee compared to baseline was accompanied by a 60-80% higher risk of progression of storage symptoms. |
| **Item is part of an intervention in the following studies** | **Outcomes of total intervention** |
| Borrie et al. (2002) ^17^  RCT in which the effect of counselling by an incontinence consultant (intervention, n = 210 of which 68 men) is compared with no care (control, n = 211 of which 66 men) in patients with incontinence. The outcome measure was the mean change in number of incontinence episodes per 24 hours , and change of incontinence material use in pieces. | Mean change in number of incontinence episodes per 24 hours  Intervention group: -1.2  Control group: -0.2  Intervention vs. control: p < 0.01.  Change of incontinence material use in pieces  Intervention group: -0.9  Control group: +0.1  Intervention vs. control: p < 0.01. |
| Brown et al (2007) and Yap et al (2009) ^3, 4^  RCT in which the effect of group sessions, supervised by a nurse and focused on self-management (intervention, n = 73 men), was compared with standard care (control, n = 67) in men with LUTS. The outcome measure was Treatment failure, defined as IPSS increase of 3 or more, use of medication for LUTS, acute urinary retention or surgery. Secondary outcomes were number of voids, nocturia episodes and LUTS severity assessed with the IPSS. | Treatment failure (intervention vs. control)  3 months: 10% vs. 42% *  6 months: 19% vs. 61% *  12 months: 31% vs. 79% *  Number of voids (intervention vs. control)  3 months: 7.1 ± 2.1 vs. 9.7 ± 3.3 *  6 months: 7.3 ± 2.2 vs. 9.0 ± 2.1 *  12 months: 7.4 ± 2.3 vs. 9.0 ± 2.6 *  Episodes of nocturia (intervention vs. control)  3 months: 1.3 ± 0.8 vs. 2.0 ± 1.4 *  6 months: 1.1 ± 0.8 vs. 1.9 ± 0.9 *  12 months: 1.0 ± 0.6 vs. 1.6 ± 1.2 *  LUTS severity assessed with IPSS (intervention vs. control)  3 months: 10.7 ± 5.9 vs. 16.4 ± 5.8 *  6 months: 10.4 ± 6.1 vs. 16.9 ± 6.4 *  12 months: 10.2 ± 6.1 vs. 15.4 ± 6.6 * |
| Chen et al. (2012) ^5^  RCT in which the effect of a self-management intervention (n = 119) was compared to regular care (n = 102) in men with BPH. Intervention was offered by means of 2 hours face to face demonstration and a follow up telephone consultation.  The outcome measure was LUTS severity assessed with the IPSS. | LUTS severity assessed with the IPSS (intervention vs. control)  Week 1: 20.6 vs. 19.4  Month 3: 14.3 ± 5.6 vs. 19.7 ± 6.5 *  Month 6: 12.0 ± 5.3 vs. 19.9 ± 6.4 * |
| Johnson et al. (2016) ^20^  RCT in which the effect of a behavioural treatment to reduce nocturia (behaviour group, n = 23) was compared with behavioural treatment with α-blockers (combination group, n = 25) and α-blockers alone (α-blocker group, n = 24) in men with nocturia. Outcome measure was change in number of nocturia episodes after 12 weeks. | Change in number of nocturia episodes after 12 weeks, compared to baseline.  Behavioural group: -1.39 ± 1.5  Combination group: -1.03 ± 1.4  α-blocker group: -0.59 ± 0.9  Difference between the groups: not statistically significant |
| **This advice is specifically mentioned in the following guidelines** | |
| - European Association of Urology (EAU) Guidelines ‘Non Neurogenic Male LUTS’ (2015) ^6^ | |
| - American Urology Association (AUA)/Society of Urodynamics, Female Pelvic Medicine and Urogenital Reconstruction (SUFU) guideline ‘OAB (non-neurogenic)’ (2014) ^7^ | |
| - American Urology Association (AUA) ‘BPH guideline’ (2014) ^8^ | |
| - Urological Association of Asia (UAA) UAA Consensus on the Management of BPH/Male LUTS (1st Edition) (2012) ^11^ | |
| - The Japanese Urological Association (JUA) Clinical guideline for Nocturia (2010) ^27^ | |
| - *NICE Guideline Lower urinary tract symptoms in men: management* ^13, 14^ | |

* Statistically significant, p <0.01

**Advice 7: alcohol management**

Explanation about the possible effect of alcohol on the bladder function. Reducing alcohol consumption.

**Level of evidence: -**

**Grade of recommendation: -**

There are no intervention studies investigating the effect of alcohol reduction in men with LUTS. However, the diuretic effect of alcohol has been studied. Alcohol use as a risk factor for developing LUTS has been studied in a number of observational studies. A systematic review from 2009 showed a protective effect of alcohol on the development of BPH (12 articles, n = 88,035). In 10 of the 12 articles, the BPH diagnosis was based on prostate surgery for LUTS. In an analysis in which only articles with LUTS were included as primary outcome (4 articles, n = 28,645), a non-significant trend was found for an increased risk of LUTS after intake of alcohol. For this, LUTS were assessed using questionnaires.

In a prospective study a significantly increased risk of developing LUTS after intake of alcohol was found.

In 3 studies this advice was part of a combined intervention.

The advice is stated in 4 guidelines.

| **Intervention studies specifically on this advice** |  |
| --- | --- |
| None |  |
| **Intervention studies effect of alcohol on the urinary tract** |  |
| Strauss et al. (1950) ^28^  Crossover study of the effect of 137 ml of whiskey (intervention) compared to 137 ml of water (control) on renal excretion in healthy subjects (sex of the participants was unspecified). Urine was collected for liquid intake and 2 and 4 hours thereafter. | 2 hours after ingestion of alcohol excretion of urine increased compared to water intake (only presented in the figure, no statistical data). Also, the total urine volume after 6 hours was higher after intake of alcohol than after intake of water (829 ml vs. 231 ml, no standard deviation or statistical tests ​​was provided). |
| Eggleton (1942) ^29^  Crossover study on the diuretic effect of different amounts of alcohol dissolved in a constant amount of cider in men. | Larger amounts of alcohol resulted in a greater total urine output (no averages and statistical values ​​given). Estimated from the figures, each extra 10 grams of alcohol provided 100 ml of extra urine output. |
| **Observational studies on this advice** | **Outcomes** |
| Parsons et al. (2009) ^30^  A systematic review of the possible association between alcohol and BPH / LUTS, in which cohort and case control studies were included. Authors included 19 studies, with a total of 120,091 men.  BPH diagnosis was based on prostate operations for micturition symptoms in 10 of the 12 articles included in the meta analysis.  LUTS were included in the meta-analysis based on the IPSS scores in all publications. | The meta-analysis (12 studies, 88,035 participants) showed that a daily intake of 36 grams of alcohol or more gave a 35% lower risk of BPH than no alcohol intake (OR 0.65, 95% CI 0.58-0.74). This effect was not found in studies with LUTS as an outcome measure (4 studies, 28,645 participants): intake of 12 grams or more alcohol per day versus no alcohol: risk of LUTS OR 1.42 (95% CI 0.77-2.64). |
| Wong et al. (2010) ^31^  Prospective cohort study on the association between lifestyle factors and LUTS in South Chinese men. | Results show that drinking 7 glasses or more alcohol per week was associated with an increased risk of having LUTS (OR 2.51; 95% CI 1.32-4.79) |
| **Item is part of an intervention in the following studies** | **Outcomes of total intervention** |
| Brown et al (2007) and Yap et al (2009) ^3, 4^  RCT in which the effect of group sessions, supervised by a nurse and focused on self-management (intervention, n = 73 men), was compared with standard care (control, n = 67) in men with LUTS. The outcome measure was Treatment failure, defined as IPSS increase of 3 or more, use of medication for LUTS, acute urinary retention or surgery. Secondary outcomes were number of voids, nocturia episodes and LUTS severity assessed with the IPSS. | Treatment failure (intervention vs. control)  3 months: 10% vs. 42% *  6 months: 19% vs. 61% *  12 months: 31% vs. 79% *  Number of voids (intervention vs. control)  3 months: 7.1 ± 2.1 vs. 9.7 ± 3.3 *  6 months: 7.3 ± 2.2 vs. 9.0 ± 2.1 *  12 months: 7.4 ± 2.3 vs. 9.0 ± 2.6 *  Episodes of nocturia (intervention vs. control)  3 months: 1.3 ± 0.8 vs. 2.0 ± 1.4 *  6 months: 1.1 ± 0.8 vs. 1.9 ± 0.9 *  12 months: 1.0 ± 0.6 vs. 1.6 ± 1.2 *  LUTS severity assessed with IPSS (intervention vs. control)  3 months: 10.7 ± 5.9 vs. 16.4 ± 5.8 *  6 months: 10.4 ± 6.1 vs. 16.9 ± 6.4 *  12 months: 10.2 ± 6.1 vs. 15.4 ± 6.6 * |
| Chen et al. (2012) ^5^  RCT in which the effect of a self-management intervention (n = 119) was compared to regular care (n = 102) in men with BPH. Intervention was offered by means of 2 hours face to face demonstration and a follow up telephone consultation.  The outcome measure was LUTS severity assessed with the IPSS. | LUTS severity assessed with the IPSS (intervention vs. control)  Week 1: 20.6 vs. 19.4  Month 3: 14.3 ± 5.6 vs. 19.7 ± 6.5 *  Month 6: 12.0 ± 5.3 vs. 19.9 ± 6.4 * |
| Johnson et al. (2016) ^20^  RCT in which the effect of a behavioural treatment to reduce nocturia (behaviour group, n = 23) was compared with behavioural treatment with α-blockers (combination group, n = 25) and α-blockers alone (α-blocker group, n = 24) in men with nocturia. Outcome measure was change in number of nocturia episodes after 12 weeks. | Change in number of nocturia episodes after 12 weeks, compared to baseline.  Behavioural group: -1.39 ± 1.5  Combination group: -1.03 ± 1.4  α-blocker group: -0.59 ± 0.9  Difference between the groups: not statistically significant |
| **This advice is specifically mentioned in the following guidelines** | |
| - European Association of Urology (EAU) Guidelines ‘Non Neurogenic Male LUTS’ (2015) ^6^ | |
| - American Urology Association (AUA) ‘BPH guideline’ (2014) ^8^ | |
| - Urological Association of Asia (UAA) Consensus on the Management of BPH/Male LUTS (1st Edition) (2012) ^11^ | |
| - The Japanese Urological Association (JUA) Clinical guideline for Nocturia (2010) ^27^ | |
| - *NICE Guideline Lower urinary tract symptoms in men: management* ^13, 14^ | |

* Statistically significant, p <0.01

**Advice 8: fluid management**

Reduce total fluid intake and / or reduce fluid intake at certain times.

**Level of evidence: -**

**Grade of recommendation: -**

There are no intervention studies investigating the effect of reducing fluid intake in men with LUTS. However, there is a small intervention study that investigated the effect of reducing or eliminating certain beverages that may be irritating to the bladder (including alcohol, caffeine, carbonated drinks and artificially sweetened beverages) in 30 women with LUTS. After reducing these drinks, these women had fewer problems with complaints and urgency, and less trouble with delaying the micturition. During this phase, the total fluid intake was also reduced, not just the irritating drinks.

In 5 studies, this advice was part of a combined intervention.

The advice is stated in 6 guidelines.

| **Intervention studies specifically on this advice** |  |
| --- | --- |
| None |  |
| **Intervention studies on this advice (in women)** | **Outcomes** |
| Miller et al. (2016) ^32^  Crossover study in which the effect of eliminating and halving potentially irritating liquids (including coffee, tea, alcohol and carbonated and / or artificially sweetened beverages) is measured in women with LUTS (n = 30). | None of the subjects was able to completely eliminate potentially irritating liquids, but all succeeded in reducing this intake. The women reported reduction of (all comparisons of baseline vs. follow-up):  Self reported urgency: 3.21 ± 0.63 vs. 2.80 ± 0.57 *  Self reported inability to postpone the micturitions: 2.91 ± 0.67 vs. 2.25 ± 0.91 *  Self reported annoyance of complaints: 29.77 ± 15.49 vs. 18.95 ± 13.4 *  Number of micturitions per 24 hours: 10.5 ± 2.64 vs. 9.2 ± 2.71 *  Possible alternative explanation for these results was the reduction of total fluid intake (2,209 ± 772 ml at baseline vs. 2,028 ± 848 ml during elimination period, p = 0.031). |
| **Item is part of an intervention in the following studies** | **Outcomes of total intervention** |
| Borrie et al. (2002) ^17^  RCT in which the effect of counselling by an incontinence consultant (intervention, n = 210 of which 68 men) is compared with no care (control, n = 211 of which 66 men) in patients with incontinence. The outcome measure was the mean change in number of incontinence episodes per 24 hours , and change of incontinence material use in pieces. | Mean change in number of incontinence episodes per 24 hours  Intervention group: -1.2  Control group: -0.2  Intervention vs. control: p < 0.01.  Change of incontinence material use in pieces  Intervention group: -0.9  Control group: +0.1  Intervention vs. control: p < 0.01. |
| Brown et al (2007) and Yap et al (2009) ^3, 4^  RCT in which the effect of group sessions, supervised by a nurse and focused on self-management (intervention, n = 73 men), was compared with standard care (control, n = 67) in men with LUTS. The outcome measure was Treatment failure, defined as IPSS increase of 3 or more, use of medication for LUTS, acute urinary retention or surgery. Secondary outcomes were number of voids, nocturia episodes and LUTS severity assessed with the IPSS. | Treatment failure (intervention vs. control)  3 months: 10% vs. 42% *  6 months: 19% vs. 61% *  12 months: 31% vs. 79% *  Number of voids (intervention vs. control)  3 months: 7.1 ± 2.1 vs. 9.7 ± 3.3 *  6 months: 7.3 ± 2.2 vs. 9.0 ± 2.1 *  12 months: 7.4 ± 2.3 vs. 9.0 ± 2.6 *  Episodes of nocturia (intervention vs. control)  3 months: 1.3 ± 0.8 vs. 2.0 ± 1.4 *  6 months: 1.1 ± 0.8 vs. 1.9 ± 0.9 *  12 months: 1.0 ± 0.6 vs. 1.6 ± 1.2 *  LUTS severity assessed with IPSS (intervention vs. control)  3 months: 10.7 ± 5.9 vs. 16.4 ± 5.8 *  6 months: 10.4 ± 6.1 vs. 16.9 ± 6.4 *  12 months: 10.2 ± 6.1 vs. 15.4 ± 6.6 * |
| Chen et al. (2012) ^5^  RCT in which the effect of a self-management intervention (n = 119) was compared to regular care (n = 102) in men with BPH. Intervention was offered by means of 2 hours face to face demonstration and a follow up telephone consultation.  The outcome measure was LUTS severity assessed with the IPSS. | LUTS severity assessed with the IPSS (intervention vs. control)  Week 1: 20.6 vs. 19.4  Month 3: 14.3 ± 5.6 vs. 19.7 ± 6.5 *  Month 6: 12.0 ± 5.3 vs. 19.9 ± 6.4 * |
| Burgio et al. (2011) ^19^  RCT in which the effect of a behavioural treatment and α-blockers (intervention, n = 73) was compared with oxybutynin + α-blockers (n = 70) in men with OAB. The outcome measures were change in 24-hour voiding frequency and nocturnal frequency, and change in LUTS severity assessed with AUA symptom index after 8 weeks. | Change in 24-hour voiding frequency after 8 weeks.  Intervention group: 11.3 ± 2.5 to 9.1 ± 2.5  Control group: 11.5 ± 2.1 to 9.5 ± 2.4  Equivalence test: p <0.01, this means the same treatment effect in both groups.  Change in nocturnal voiding frequency after 8 weeks.  Intervention group: -0.70 ± 0.72  Control group: -0.32 ± 1.28  Intervention vs. control: p<0.01  Change in LUTS severity assessed with AUA symptom index after 8 weeks.  Intervention group: -3.4 ± 4.6  Control group: -3.2 ± 5.6  Intervention vs. control: not statistically significant |
| Johnson et al. (2016) ^20^  RCT in which the effect of a behavioural treatment to reduce nocturia (behaviour group, n = 23) was compared with behavioural treatment with α-blockers (combination group, n = 25) and α-blockers alone (α-blocker group, n = 24) in men with nocturia. Outcome measure was change in number of nocturia episodes after 12 weeks. | Change in number of nocturia episodes after 12 weeks, compared to baseline.  Behavioural group: -1.39 ± 1.5  Combination group: -1.03 ± 1.4  α-blocker group: -0.59 ± 0.9  Difference between the groups: not statistically significant |
| **This advice is specifically mentioned in the following guidelines** | |
| - European Association of Urology (EAU) Guidelines ‘Non Neurogenic Male LUTS’ (2015) ^6^ | |
| - American Urology Association (AUA)/Society of Urodynamics, Female Pelvic Medicine and Urogenital Reconstruction (SUFU) guideline ‘OAB (non-neurogenic)’ (2014) ^7^ | |
| - American Urology Association (AUA) ‘BPH guideline’ (2014) ^8^ | |
| - Dutch college of general practitioners guideline Male LUTS (2013) ^9, 10^ | |
| - Urological Association of Asia (UAA) UAA Consensus on the Management of BPH/Male LUTS (1st Edition) (2012) ^11^ | |
| - The Japanese Urological Association (JUA) Clinical guideline for Nocturia (2010) ^27^ | |
| - *NICE Guideline Lower urinary tract symptoms in men: management* ^13, 14^ | |

* Statistically significant, p <0.01

**Advice 9: exercise advice**

Reducing overweight / obesity and advice to exercise more.

**Level of evidence: -**

**Grade of recommendation: -**

There are no intervention studies investigating the effect of more exercise and weight loss on LUTS. However, there is a systematic review of 8 observational studies (n = 35,675) in which the relationship between physical activity and LUTS / BPH has been investigated. The meta-analysis showed that an active lifestyle reduces the risk of developing LUTS.

In another systematic review the relationship between BMI and LUTS / BPH has been investigated. The meta-analysis of 15 studies (n = 49,181) showed that a higher BMI gives a greater chance of LUTS / BPH. After exclusion of articles with BPH as an outcome measure, no statistically significant relationship was found between BMI and LUTS (4 studies, n = 29,675).

Two more recent prospective studies showed that higher BMI is associated with a higher chance of developing new LUTS and progression of existing LUTS.

In no study was the recommendation for more exercise part of a combined intervention

The advice is stated in 4 guidelines.

| **Intervention studies specifically on this advice** |  |
| --- | --- |
| None |  |
| **Observational studies on this advice** | **Outcomes** |
| Parsons et al. (2008) ^33^  Systematic review of 11 observational studies that examined the relationship between physical activity and BPH and LUTS. 8 studies of this were used in the meta-analysis (N = 35,675 men). | Compared with sedimentary lifestyles, men who have moderate and strong physical activity had a smaller chance of developing LUTS: OR 0.74 (95% CI 0.60-0.92) and 0.74 (95% CI 0.59-0.92), respectively. Men who perform light physical activity had a smaller chance of developing LUTS than sedimentary men: OR 0.70 (95% CI 0.44-1.13). |
| Wang et al. (2012) ^34^  Systematic review of studies published before December 2010 in which the relationship between BMI and BPH and LUTS was investigated.  BPH was defined in 7 studies by operation, in 2 studies by prostate volume and in 2 studies by clinical diagnosis.  LUTS was defined by means of IPSS or AUA SI in 4 articles. | Meta analysis of 15 studies with 49,181 participants showed an association between BMI and probability of BPH / LUTS (OR 1.23, 95% CI 1.03-1.48) when BPH and LUTS were included as an outcome measure.  From 4 studies with 29,675 participants who only had LUTS as outcome measure, there was no significant relationship between BMI and the chance of LUTS (OR 1.11; 95% CI 0.84-1.47). |
| Mondul et al. (2014) ^35^  Prospective study of the association between overweight and the development of LUTS (n = 4,088 men) and overweight and the progression of LUTS (n = 1,691 men). | Men with a BMI of ≥35 have a higher risk of developing new LUTS (Hazard ratio 1.61, 95% CI 1.31-1.99) than men with a BMI of 23 to 25.  Men with a BMI of ≥35 are more likely to experience progression of LUTS (HR 1.44, 95% CI 1.04-2.00) than men with a BMI of 23 to 25. |
| Parsons et al. (2011) ^36^  Prospective observational study investigating the association between obesity and LUTS and between physical activity and LUTS (n = 1,695). | Men with overweight (BMI 25-30) and men with obesity (BMI> 30) have, respectively, 29% (OR 1.29, 95% CI 1.00-1.68) and 41% (OR 1.41, 95% CI 1.03-1.93) a higher risk to develop LUTS than men with normal weight (BMI <25).  Men from the highest quartile of measured physical activity (PASE score) were 29% less likely to develop LUTS (OR 0.71, 95% CI 0.53-0.97) than men who led sedentary life. For those who walked daily, this was 20% (OR 0.8, 95% CI 0.65-0.98). |
| **This advice is specifically mentioned in the following guidelines** | |
| - American Urology Association (AUA)/Society of Urodynamics, Female Pelvic Medicine and Urogenital Reconstruction (SUFU) guideline ‘OAB (non-neurogenic)’ (2014) ^7^ | |
| - Dutch college of general practitioners guideline Male LUTS (2013) ^9, 10^ | |
| - Urological Association of Asia (UAA) UAA Consensus on the Management of BPH/Male LUTS (1st Edition) (2012) ^11^ | |
| - The Japanese Urological Association (JUA) Clinical guideline for Nocturia (2010) ^27^ | |

**References**

1. Wolters R, Wensing M, Van Weel C et al: The effect of a distance-learning programme on patient self-management of Lower Urinary Tract Symptoms (LUTS) in general practice: a randomised controlled trial. Eur Urol 2004; **46:** 95.

2. Brown CT, O'Flynn E, Van Der Meulen J et al: The fear of prostate cancer in men with lower urinary tract symptoms: should symptomatic men be screened?. BJU Int 2003; **91:** 30.

3. Brown CT, Yap T, Cromwell DA et al: Self management for men with lower urinary tract symptoms: randomised controlled trial. BMJ 2007; **334:** 25.

4. Yap TL, Brown C, Cromwell DA et al: The impact of self-management of lower urinary tract symptoms on frequency-volume chart measures. BJU Int 2009; **104:** 1104.

5. Chen Y, Zhang X, Hu X et al: The potential role of a self-management intervention for benign prostate hyperplasia. Urology 2012; **79:** 1385.

6. Gratzke C, Bachmann A, Descazeaud A et al: EAU Guidelines on the Assessment of Non-neurogenic Male Lower Urinary Tract Symptoms including Benign Prostatic Obstruction. Eur Urol 2015; **67:** 1099.

7. Gormley EA, Lightner DJ, Burgio KL et al: Diagnosis and treatment of overactive bladder (non-neurogenic) in adults: AUA/SUFU guideline. The Journal of Urology 2014; .

8. McVary KT, Roehrborn CG, Avins AL et al: Update on AUA guideline on the management of benign prostatic hyperplasia. J Urol 2011; **185:** 1793.

9. Blanker MH, Breed SA, van der Heide WK et al: NHG-Standaard Mictieklachten bij mannen. Huisarts Wet 2013; 114-122.

10. Blanker MH, Klomp MA, van den Donk M et al: Summary of the NHG practice guideline 'Lower urinary tract symptoms in men'. Ned Tijdschr Geneeskd 2013; **157:** A6178.

11. Takeda M, Rahman MA, Salam MA et al: UAA Consensus on the Management of BPH/Male LUTS (1st Edition) 2012; .

12. Abrams P, Chapple C, Khoury S et al: Evaluation and treatment of lower urinary tract symptoms in older men. J Urol 2009; **181:** 1779.

13. National Clinical Guideline Centre (NICE): Lower urinary tract symptoms in men: management (Clinical guideline CG97) 2015; **CG97:** .

14. Jones C, Hill J, Chapple C et al: Management of lower urinary tract symptoms in men: summary of NICE guidance. BMJ 2010; **340:** c2354.

15. Dorey G, Speakman M, Feneley R et al: Pelvic floor exercises for treating post-micturition dribble in men with erectile dysfunction: a randomized controlled trial. Urol Nurs 2004; **24:** 490.

16. Paterson J, Pinnock CB, Marshall VR: Pelvic floor exercises as a treatment for post-micturition dribble. Br J Urol 1997; **79:** 892.

17. Borrie MJ, Bawden M, Speechley M et al: Interventions led by nurse continence advisers in the management of urinary incontinence: a randomized controlled trial. CMAJ 2002; **166:** 1267.

18. Ruiz JG, Tunuguntla R, Cifuentes P et al: Development and pilot testing of a self-management internet-based program for older adults with overactive bladder. Urology 2011; **78:** 48.

19. Burgio KL, Goode PS, Johnson TM et al: Behavioral versus drug treatment for overactive bladder in men: the Male Overactive Bladder Treatment in Veterans (MOTIVE) Trial. J Am Geriatr Soc 2011; **59:** 2209.

20. Johnson TM,2nd, Burgio KL, Redden DT et al: Effects of behavioral and drug therapy on nocturia in older incontinent women. J Am Geriatr Soc 2005; **53:** 846.

21. Bernards AT, Berghmans BC, Slieker-Ten Hove MC et al: Dutch guidelines for physiotherapy in patients with stress urinary incontinence: an update. Int Urogynecol J 2014; **25:** 171.

22. Mattiasson A, Blaakaer J, Hoye K et al: Simplified bladder training augments the effectiveness of tolterodine in patients with an overactive bladder. BJU Int 2003; **91:** 54.

23. Bryant CM, Dowell CJ, Fairbrother G: Caffeine reduction education to improve urinary symptoms. Br J Nurs 2002; **11:** 560.

24. Nussberger J, Mooser V, Maridor G et al: Caffeine-induced diuresis and atrial natriuretic peptides. J Cardiovasc Pharmacol 1990; **15:** 685.

25. Lohsiriwat S, Hirunsai M, Chaiyaprasithi B: Effect of caffeine on bladder function in patients with overactive bladder symptoms. Urol Ann 2011; **3:** 14.

26. Maserejian NN, Wager CG, Giovannucci EL et al: Intake of caffeinated, carbonated, or citrus beverage types and development of lower urinary tract symptoms in men and women. Am J Epidemiol 2013; **177:** 1399.

27. Committee for Establishment of the Clinical Guidelines for Nocturia of the Neurogenic Bladder Society: Clinical guidelines for nocturia. Int J Urol 2010; **17:** 397.

28. Strauss MB, Rosenbaum JD, Nelson WP,3rd: The effect of alcohol on the renal excretion of water and electrolyte. J Clin Invest 1950; **29:** 1053.

29. Eggleton MG: The diuretic action of alcohol in man. J Physiol 1942; **101:** 172.

30. Parsons JK, Im R: Alcohol consumption is associated with a decreased risk of benign prostatic hyperplasia. J Urol 2009; **182:** 1463.

31. Wong SY, Woo J, Leung JC et al: Depressive symptoms and lifestyle factors as risk factors of lower urinary tract symptoms in Southern Chinese men: a prospective study. Aging Male 2010; **13:** 113.

32. Miller JM, Garcia CE, Hortsch SB et al: Does Instruction to Eliminate Coffee, Tea, Alcohol, Carbonated, and Artificially Sweetened Beverages Improve Lower Urinary Tract Symptoms?: A Prospective Trial. J Wound Ostomy Continence Nurs 2016; **43:** 69.

33. Parsons JK, Kashefi C: Physical activity, benign prostatic hyperplasia, and lower urinary tract symptoms. Eur Urol 2008; **53:** 1228.

34. Wang S, Mao Q, Lin Y et al: Body mass index and risk of BPH: a meta-analysis. Prostate Cancer Prostatic Dis 2012; **15:** 265.

35. Mondul AM, Giovannucci E, Platz EA: A prospective study of obesity, and the incidence and progression of lower urinary tract symptoms. J Urol 2014; **191:** 715.

36. Parsons JK, Messer K, White M et al: Obesity increases and physical activity decreases lower urinary tract symptom risk in older men: the Osteoporotic Fractures in Men study. Eur Urol 2011; **60:** 1173.
