# Supplementary material for: Development of an online personalized self‐management intervention for men with uncomplicated LUTS
Source: Neurourol Urodyn. 2019 May 20;38(6):1685–91. doi: 10.1002/nau.24040 (PMC6851544; doi:10.1002/nau.24040)
Supplement: Supplementary file 3 — Supporting information [file NAU-38-1685-s003.docx]

**Supplementary file 2 Detailed description of the evidence base for the nine advices found in the scoping review**

In the survey among physicians, the advice, level of evidence, grade of recommendation and the evidence summary were shown. The additional information presented in the Table was visible if participants selected the option to read more.

Please note that the NICE guideline was not included in the data provided in the survey amongst the physicians, despite the fact that it had served as an important source of information in the primary search. We have added this guideline to the Tables as it should have been presented.
